# Comparative Life Cycle Analysis of Concrete and Composite Bridges Varying Steel Recycling Ratio

**DOI:** 10.3390/ma14154218

**Published:** 2021-07-28

**Authors:** David Martínez-Muñoz, Jose V. Martí, Víctor Yepes

**Affiliations:** Institute of Concrete Science and Technology (ICITECH), Universitat Politècnica de València, 46022 Valencia, Spain; jvmartia@cst.upv.es (J.V.M.); vyepesp@cst.upv.es (V.Y.)

**Keywords:** life cycle assessment, sustainability, structures, ReCiPe, environment, bridges

## Abstract

Achieving sustainability is currently one of the main objectives, so a consensus between different environmental, social, and economic aspects is necessary. The construction sector is one of the main sectors responsible for environmental impacts worldwide. This paper proposes the life cycle assessment (LCA) and comparison of four bridge deck alternatives for different span lengths to determine which ones are the most sustainable solutions. The ReCiPe method is used to conduct the life cycle analysis, by means of which the impact value is obtained for every alternative and span length. The Ecoinvent 3.3 database has been used. The life cycle has been divided into four phases: manufacturing, construction, use and maintenance, and end of life. The associated uncertainties are considered, and the results are shown in both midpoint and endpoint approaches. The results of our research show that for span lengths less than 17 m, the best alternative is the prestressed concrete solid slab. For span lengths between 17 and 25 m, since the box-girder solution is not used, then the prestressed concrete lightened slab is the best alternative. For span lengths between 25 and 40 m, the best solution depends on the percentage of recycled structural steel. If this percentage is greater than 90%, then the best alternative is the composite box-girder bridge deck. However, if the percentage is lower, the cleanest alternative is the prestressed concrete box-girder deck. Therefore, the results show the importance of recycling and reusing structural steel in bridge deck designs.

## 1. Introduction

Over the last few years, awareness of the consequences of the consumption of raw materials and the emissions of various processes has risen. Society has realized that if we continue with the uncontrolled consumption of resources, our current actions will compromise the future of the planet. For this reason, the sustainable development concept appeared, a term that was introduced in 1987 by the Brundtland Commission, defining it as *“development that meets the needs of the present without compromising the ability of future generations to meet their own needs”* [1]. Since then, a significant effort has been invested to achieve cleaner production processes for known materials and the development of new materials with the same characteristics but fewer contaminants.

Construction is one of the most carbon-intensive industries [2,3], and in terms of CO_2_ emissions, its cement requirements alone produce 5% of the total emissions [4]. Furthermore, construction contributes to environmental pollution [5]. This negative contribution is mainly produced by cement and concrete production [4,6]. The impact of these activities is produced by their energy consumption, and in the construction sector, concrete is one of the most important materials used in buildings. Due to this circumstance, concrete consumption, and therefore the associated pollution, will increase over the next years [7]. Because of this, human activities must be optimized in terms of material consumption and emissions to ensure more sustainable processes that will not compromise the environment as much.

Due to the importance of achieving this objective, many researchers have been studying current construction processes in order to improve and optimize their sustainability. Researchers have studied the emissions produced by concrete projects [8,9,10] or construction procedures [11,12,13]. Other studies have focused on the optimization of concrete structures such as prestressed bridges [14,15] and earth-retaining walls [16,17,18,19]. Other researchers have studied CO_2_ fixation by carbonation processes and their influence on the emissions [20,21] and the concrete recycling ratio [22,23,24].

However, to study the environmental impact, life cycle analysis (LCA) is performed. This is a powerful and versatile method capable of evaluating any type of construction or process [25] or the materials used therein [26,27,28], from concrete and earth retaining walls [29,30] to optimal bridge decks [31], house structures [32], and facades [33,34]. However, some reviews state that there is a lack of LCA in steel–concrete composite bridges [35].

The aim of this study is to carry out analyses of the life cycle of four bridges: prestressed concrete solid slab (PCSS), prestressed concrete lightened slab (PCLS), prestressed concrete box-girder (PCBG), and steel‒concrete composite box-girder (CBG). The aim is to determine which of them, depending on the span length, has the lowest environmental impact [22,23,36]. Additionally, a sensitivity analysis is carried out to evaluate the impact of the life cycle of a composite bridge depending on its steel recycling ratio in order to study the feasibility of this structural type compared to concrete alternatives. This allows us to provide a broader approach and to make a comparison of the amount of recycled steel that has been used in the manufacturing processes. Steel manufacturing comprises two main production methods: basic oxygen furnace (BOF) and electric arc furnace (EAF). In both processes, iron is combined with steel scrap, which is the product that is obtained by the steel recycling process. In EAF production, the percentage of steel scrap (recycled steel) used is between 90 and 100%, while in the BOF production process, the percentage of steel scrap (recycled steel) is reduced to 10‒30% [37]. The rates of BOF and EAF and, in consequence, the rate of recycled steel used for steelmaking depend largely on the technological development of countries. Therefore, this makes this study useful not only for countries with a high technological development in the steelmaking process but also for other countries where steel contains a smaller amount of recycled raw material.

## 2. Materials and Methods

The life cycle analysis (LCA) method consists of obtaining the environmental impact of an activity, evaluating the potential contribution of the processes that make up that product. These processes together encompass all the activities required to complete the main product. The procedures begin by obtaining the raw material and end with the waste management. The LCA of the bridge decks has been carried out according to ISO 14040:2006 [38]. It comprises four phases to obtain the assessment: definition of goal and scope, inventory analysis, impact assessment, and interpretation of the results. The life cycle impact assessment (LCIA) that has been chosen for this research is the ReCiPe 2008 method [39]. The database used to obtain the environmental impact information is Ecoinvent v3.3.

### 2.1. Goal and Scope Definition

The main goal of this research is to compare, from the environmental point of view, four different bridge deck types. The structural system selected is a continuous beam, and the analyses have been carried out on six span lengths: 15, 20, 25, 30, 35, and 40 m. The purpose of this research is to compare different deck types with different span lengths to evaluate the differences between them, and the LCA method makes it possible to obtain a quantitative assessment of the different solutions proposed. Pang et al. [40] affirm that there are three main reasons for performing an LCA analysis on bridges: comparison of different alternatives, comparison of different bridge component alternatives, and comparison of new material with conventional material. To compare different bridge alternatives, these have to be similar in terms of load capacities, deck dimensions, and span if all the alternatives are in the same geographical area. If they are not, then it is necessary to take into account other conditions such as the geotechnical information of the ground, the seismicity of the building location, and the corrosion capacity of the environment, among others. In this study, the same location has been considered for all the alternatives.

#### 2.1.1. Bridge Deck Type Selection

**Figure 1:**
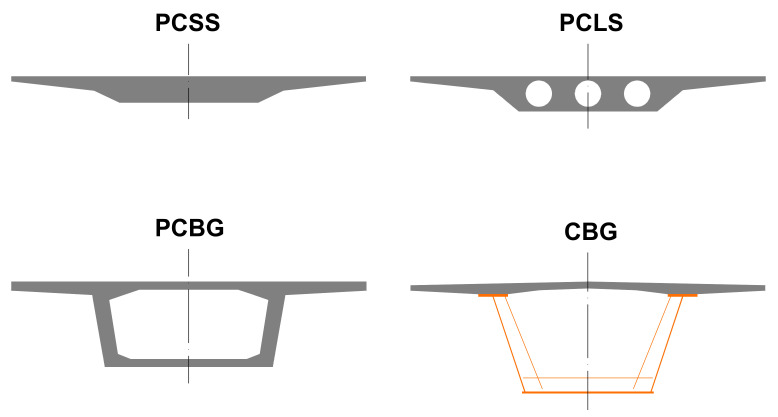
Bridge deck cross sections.

Bridges are very important infrastructures that allow society to avoid obstacles and enable users to close the gap between two points. Furthermore, these structures have a direct impact on society in terms of their economic, social, and environmental role. For beam bridges, the most important part is the deck, because it has to resist the stresses produced by the traffic loads. The deck type depends on different conditionings: functional, constructive, economic, and environmental, among others. In this paper, four deck types have been compared: prestressed concrete solid slab (PCSS), prestressed concrete lightened slab (PCLS), prestressed concrete box-girder (PCBG), and composite box-girder (CBG). Figure 1 presents a sketch of each of the alternatives.

The use of these decks depends on the span length. On the one hand, continuous slab depth decks are used for lengths between 5 and 50 m, but the usual range of application is from 15 to 35 m. However, the box-girder bridges have lengths between 25 and 125 m, and the most used range is 35‒80 m. In box-girder bridges, two main materials are used to form the resistant section of the bridge: concrete, which can be reinforced or prestressed, and steel. The choice of material may depend on various factors, including the environment to which the structure is exposed, the road alignment, and geotechnical constraints, among others.

Slab bridges are usually used for shorter distances, because for greater lengths, the amounts of concrete, passive reinforcing steel (PRS), and active reinforcing steel (ARS) increase to a large extent, which translates into a higher cost, which is even more acute in PCSS. On the other hand, box-girder bridges can be used for greater lengths due to the ease of increasing their bridge deck depth, taking advantage of the mechanical arm increase and making the most of the mechanical characteristics of the materials. This study focuses on the comparison of these four bridge deck types with span lengths from 15 to 40 m, assessing them from the environmental point of view. This selection of alternatives comprises two slab and two box-girder decks, providing a wide range of choice and an estimation of the environmental cost of the structures for structural engineers and designers, depending on the dimensions of the structure.

#### 2.1.2. Phases of the Analysis

Four stages are defined to assess the bridge’s life cycle. To carry out the complete analysis of the structure, the processes that encompass all the activities should be considered, starting with the design, going through the manufacturing and the construction of the structure, and finally, the demolition and collection of the used materials. To consider all these activities, the life cycle of the structure is divided into four phases: manufacturing, construction, use and maintenance, and end of life, depending on the moment at which every activity is carried out. In this paper we have focused on PCSS, PCLS, PCBG, and CBG deck types, but it could be used for all bridge deck types by making minor modifications.

##### Manufacturing

**Figure 2:**
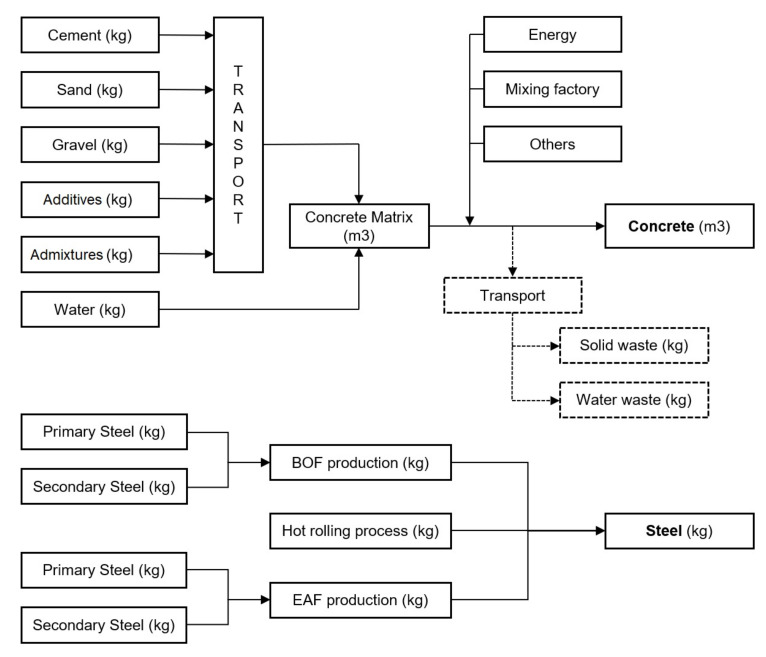
Concrete and steel manufacturing processes.

The manufacturing stage includes all activities needed to produce the materials that will be used for the resistant section, since the raw materials are extracted to be ready for use in the construction phase. The most widely used materials in bridge structures are concrete and steel. Databases usually refer to products that allow for the modeling of these materials, but it is possible to create a new product with real manufacturing processes and distances or in the case of concrete, with different dosages. The general processes to obtain one cubic meter of concrete and one kilogram of steel are shown in Figure 2.

The concrete matrix is created from different components that allow the quantity of each product that forms the concrete matrix to be controlled. Furthermore, it permits the control of the distance that every raw material is transported to the concrete manufacturing factory, allowing the study to be more specific, depending on the location where the concrete is created. Once all the materials of the concrete matrix are brought together, to simulate the concrete mixing, another process is created, including the concrete matrix along with the energy, the mixing factory, or other activities that are needed to create one cubic meter of the final concrete product.

To produce a cubic meter of concrete, the mass of the final product and the wastes produced in the process must be considered. Marceau et al. [41] concluded that for the production of one cubic meter of concrete, the solid waste is 24.5 kg and the wastewater is 0.0348 m^3^, the solid waste being small amounts of concrete. The real amount of each material that forms the concrete matrix can be calculated in Equations (1)–(5) [31].

(1) Totalsolid=Cement+Gravel+Sand

(2) $$Primary\;cement=Cement+\left(\frac{Cement}{Totalsolid}\right)\cdot Waste\;concrete$$

(3) $$Primary\;gravel=Gravel+\left(\frac{Gravel}{Totalsolid}\right)\cdot Waste\;concrete$$

(4) $$Primary\;sand=Sand+\left(\frac{Sand}{Totalsolid}\right)\cdot Waste\;concrete$$

(5) Primarywater=Water+Wastewater

Steel manufacturing comprises two main production methods: basic oxygen furnace (BOF) and electric arc furnace (EAF). In both processes, iron is combined with steel scrap, which is the product that is obtained by the steel recycling process. In EAF production, the percentage of steel scrap (recycled steel) used is between 90 and 100%, while in the BOF production process, the percentage of steel scrap (recycled steel) is reduced to 10‒30% [37]. The use of steel scrap has a direct relation with the environmental impact, and for this reason, the EAF and BOF have very different impacts. The ratio of steel scrap used for BOF and EAF production is known, so by controlling the EAF and BOF ratio to produce a kilogram of steel, the quantity of recycled steel for each steel product can be controlled in the manufacturing processes. The BOF and EAF waste production is considered in the product manufacturing part of the database.

The steel recycling ratio is especially important for steel and steel‒concrete composite bridges. Because of the great amounts of steel used in their construction, slight variations in the steel recycling ratio produce great differences in the environmental impact of the bridge, as it is produced in general with a great amount of steel. From this point of view, it is important to distinguish between structural and rebar steel, as the USA steel recycling ratio for rebar steel is 71%, while the structural steel recycling ratio is 98% [42]. The difference between the recycling ratios of these two types of steel is occasioned by the difficulty of separating rebar steel from the concrete, because of which the recycling ratio for rebar steel is lower. Furthermore, the separation between the EAF and BOF steel production processes allows the specific steel recycling ratio of the study area to be introduced [29].

##### Construction

The construction phase includes all the activities that are necessary to build the bridge, considering the machinery, depending on the chosen construction method and the location of the structure. The construction method must be defined at this stage. At this stage, the formwork, scaffolding, vibrators, and concrete pouring must be considered. In addition, for steel and steel‒concrete composite bridges, the processes of welding the different parts that have not been considered in the manufacturing phase must be introduced. The construction method is introduced in the LCA model through the diesel consumption of the machinery obtained from the manufacturer’s data, the literature, or other databases.

##### Use and Maintenance

The use and maintenance stage contains all the activities that will be needed throughout the life of the structure. These activities can be classified into three different categories: maintenance activities, CO_2_ fixation, and traffic detour. To carry out the different maintenance activities, the partial or total closure of the bridge may be necessary. If closure of the bridge is necessary, it implies that the vehicles will need to take an alternative route to reach their destination. This increase of the distance is translated into an increment of the environmental impact. The traffic detour impact is affected by different factors such as the location of the structure, the ratio of heavy vehicles, and the detour distance.

Authors have two different options to handle the maintenance stage. On the one hand, researchers assess the maintenance operations through a literature review to consider these operations [22,23,43]. On the other hand, different possible scenarios have been considered to analyze which one has the lowest environmental impact [40]. If the closure of the bridge is necessary for the maintenance activities, and their duration is defined, then this activity will be considered by introducing the processes that simulate those activities. These processes depend on the material used in the design of the bridge. The maintenance of steel bridges depends on the type of steel; if the steel is resistant to corrosion, this operation will be irrelevant, but if the steel needs to be treated for corrosion, this treatment will be repeated along the bridge life. For concrete bridges, these operations include the demolition of the external layer and their replacement with a reparation mortar. All these operations are considered by introducing the materials necessary for the repair, the diesel consumption of the machinery, and the emissions produced by the traffic detour if it occurs.

On the other hand, studies have concluded that concrete can fix CO_2_ through carbonation [8,20,44]. Carbonation is one of the principal damage mechanisms of reinforced concrete bridges, and it is determined by three main factors [21]: the w/b ratio, the concentration of CO_2_ in the surrounding air, the specific climate conditions, and the depth of embedded steel. Carbonation damages the concrete structure, but if we focus on the environmental impact, carbonation reduces the structure’s environmental impact. Lagerblad et al. [45] studied the CO_2_ fixed by carbonation during the life-cycle based on Fick’s first law. Equation 6 allows the fixed CO_2_ to be calculated, in which *k* is the carbonation coefficient, *t* is the service life, *A* is the exposed area of concrete, *r* is the ratio of CaO that is going to become carbonated, *C* is the content of cement in one cubic meter of concrete, *k* is the content of clinker in the cement, *L* is the content of CaO in the clinker, and ϵ is the molecular weight ratio of $CO_{2}/CaO$. This equation is simplified grouping the constants. Lagerblad et al. [45] consider that *r* takes the value of 0.75 and *L* of 0.65 and assume that ϵ takes the value of 0.7857. Clearing out the equation with these constants, the expression changes to (7). García-Segura et al. [21] state that concrete structures can fix CO_2_ along their service life.

(6) $$CO_{2}fixed\;\left(kg\right)=\frac{k\left(\frac{mm}{\sqrt{year}}\right)\cdot \sqrt{t(year)}}{1000}\cdot A\left(m^{2}\right)\cdot r\cdot C\left(\frac{kg}{m^{3}}\right)\cdot k\left(\%\right)\cdot L\left(\%\right)\cdot \epsilon$$

(7) $$CO_{2}fixed\;\left(kg\right)=0.383\cdot \frac{k\left(\frac{mm}{\sqrt{year}}\right)\cdot \sqrt{t(year)}}{1000}\cdot A\left(m^{2}\right)\cdot C\left(\frac{kg}{m^{3}}\right)\cdot k\left(\%\right)$$

##### End of Life

The end of life stage includes all the activities related to the dismantling of the structure, i.e., with the processes that occur when the life of the structure has ended. The principal processes involved in that stage of the bridge life are the machinery used to carry out the demolition of the structure and the transport and the treatment of the generated waste products. Consequently, it is necessary to define the distances between the building location and the landfill or the waste treatment plants. There are three main possibilities for the waste materials: to reuse them, to recycle them, or to dispose of them in a landfill, depending on their characteristics. In this case study, and generally in bridges, the most commonly used materials are concrete and steel, and depending on the needs of the society of the region studied, there will be several possibilities for the waste treatment.

The steel recycling ratio has been studied by many researchers. Hammervold et al. [43] considered a 100% steel recycling ratio, while other authors such as Du et al. [23] and Hettinguer et al. [24] considered a lower value. Penadés-Plà et al. [46] considered the Spanish average steel recycling ratio of 71%. Other authors use the average value of larger areas of study. As you can see above, the steel recycling ratio depends on the location of the construction, and it is possible to refine the assessment of the steel used in the LCA model by controlling the ratio.

The concrete case is different from steel, because it can be recycled and reused with ease, especially in bridges. The Spanish concrete regulations recommend using at most 20% of concrete recycled coarse aggregates to produce new concrete [47]. Different concrete recycling ratios are considered [22,23,24]. As described before, the carbonation processes of concrete are carried out. If all the concrete is crushed [21], the surface available to perform the carbonation processes increases; therefore, the carbonation of the all concrete volume can be produced. Lagerblad [45] states the coefficient for concrete carbonation depending on concrete’s strength. In this study, two types of concrete have been used, with 30 and 40 MPa strengths. The carbonation coefficient (*k*) is 1.5 mm/year^0.5^, 4 mm/year^0.5^, 6 mm/year^0.5^, 0.75 mm/year^0.5^, and 1 mm/year^0.5^, depending on whether the concrete is exposed, sheltered, indoors, wet, or buried, for 30 MPa concrete strength, and 1 mm/year^0.5^, 2.5 mm/year^0.5^, 3.5 mm/year^0.5^, 0.5 mm/year^0.5^, and 0.75 mm/year^0.5^ for 40 MPa concrete strength. The crushed concrete aggregate is assumed to have a 10 mm diameter.

#### 2.1.3. Functional Unit

The study has been realized considering a square meter as the functional unit to enable the comparison of the different bridges. It is necessary to carry out a comparison between bridges to consider other factors such as the geotechnical parameters of the soil, seismic conditions, or contour restraints. If the location of the studied bridges is different, then the impact can differ depending on the processes used in the manufacturing of the materials. Another possibility is to consider the linear meter as the functional unit, as Penadés-Plà et al. [46] did. To compare the linear meter with the square meter, the values of the parameters must be divided by the deck width.

### 2.2. Inventory Analysis

The inventory analysis consists of the data collection of all the materials and energy consumption that are needed to develop all the processes involved in the bridge life cycle; in this case, all the values are referred to a square meter of bridge. These processes produce an output in terms of emissions to the environment, and the consideration of the output of every process together gives the environmental impact associated with the product that is being assessed.

#### Software

The model has been developed with the OpenLCA software from GreenDelta. This is an open source program that allows LCA applications to be performed, especially for the scientific community [48]. Furthermore, this software allows the introduction of the uncertainty attached to the processes previously imported from a database.

The database used to import the processes was Ecoinvent [49] in its version 3.3. This database was selected for this study because of its scientific reliability and constant updating [50].

### 2.3. Uncertainty

Uncertainty appears in LCA analyses due to the differences between the processes that are implemented in the database and the real ones [51]. These differences are caused by different factors, but the most important are the geographical location [52] and the time period over which the data were collected. For instance, it is not the same producing a kilogram of steel in Germany or in Spain, because the technology of the production process or the distances between the quarry and the facilities differ, and the manufacturing processes of steel in Spain in 2000 or in 2017 cannot be considered the same. These variations of location and time will introduce uncertainty in the processes.

To accommodate this uncertainty, the pedigree matrix [53] has been used. This method allows uncertainty factors to be introduced by means of five indicators: reliability, completeness, temporal correlation, geographical correlation, and further technological correlation. In addition, a basic uncertainty factor will be considered depending on the nature of the processes [49].

### 2.4. Bridge Deck Design

In Table 1, the quantity of materials per square meter has been provided. The amounts of materials for the PCSS and PCLS bridge decks have been obtained from the study by Yepes et al. [54]. The materials used to define the PCBG and CBG deck alternatives have been obtained from the instruction “Obras de paso de nueva construcción” of the Spanish Ministry of Public Works [55].

Table 2 shows the dosage of 30 and 40 MPa strength concrete used for this study. The PCSS, PCLS, and PCBG decks have been designed with HP-40 prestressed concrete, while for the CBG bridge deck, HA-30 reinforced concrete has been considered. The biggest difference in the use of materials is that in the CBG alternative, a steel‒concrete composite structure, the structural steel beam that supports the slab, is added.

#### 2.4.1. Life Cycle Model Description

The life cycle model comprises four stages, the processes considered for the modeling of the decks have been obtained mainly from the Ecoinvent database, and those that were not included there have been generated, such as some types of machinery that have been modeled by their diesel consumption considering their operation times.

##### Manufacturing

In the production phase, all the processes to produce materials have been included. In addition, the transport of the materials to the construction site has been considered, where the distances between the facilities and the building location are 30 km for concrete and 150 km for both the structural and rebar steels. Two types of concrete have been introduced depending on the deck type. Concrete of 30 MPa strength has been introduced directly from the Ecoinvent database, while the 40 MPa strength concrete process has been created as shown in Figure 2.

Steel production has been considered, creating two different steel production processes to consider the differences between the steel recycling ratio of the rebar and the structural steels. Ecoinvent’s BOF process considers 19% of steel scrap (recycled steel), while the EAF process considers 100% of steel scrap. If the steel scrap amount is known, it is possible to control the total steel recycling ratio for the rebar and the structural steels. For the former, a 71% steel recycling ratio has been considered, while for the structural steel, many different recycling ratios have been determined to study their different impacts. Those ratios are 71% (CBG_71), 90% (CBG_90), and 98% (CBG_98). This varying of the steel recycling ratios has been considered in order to reflect differences between countries reusing materials, because in developing countries, policies that consider reuse are lower [56].

Furthermore, the CBG bridge deck needs to take into account the welding of the steel sheets in the manufacturing process, so this has been introduced in the CBG model considering the Ecoinvent database process.

##### Construction

Construction was considered to be in situ. The activities considered in this stage are those related with concrete pouring and vibrating, the assembly of the different steel parts of the CBG, and the handling of the active reinforcement steel. A concreting with no special concrete curing requirements has been considered. The machinery is modeled introducing the diesel consumption data, which have been obtained from the Bedec database [57]. The diesel consumption is 123.42 MJ of energy per cubic meter of concrete and 10.2 MJ per kg of active reinforcement steel. The CO_2_ emissions are 32.24 kg and 2.62 kg, respectively.

##### Use and Maintenance

For the use and maintenance phase, it has been considered that traffic detours are not necessary to carry out the maintenance operations and that only the concrete needs to be maintained because the steel that has been considered is a weathering steel that does not need maintenance. The machinery for the maintenance was estimated considering two different periods of maintenance. The machinery consumption contemplated in this phase of the life cycle is 584.28 MJ, and the CO_2_ emissions are 46.58 kg of CO_2_ per square meter repaired.

##### End of Life

At this stage, the activities related to the demolition and transport to landfill have been introduced in the LCA model. On the one hand, for the concrete elements, the machinery needed for their demolition has been considered. In addition, to be able to consider that all the concrete is carbonated, the crushing process has been included. On the other hand, only the transportation to the landfill has been reflected in the model because the recycling process of the steel has already been taken into account in the manufacturing process. For the CBG bridge deck alternative, steel sheet cutting with a flame cutting process has been considered.

### 2.5. Impact Assessment

The impact assessment consists of converting the impact of the processes considered to model the life cycle with an indicator that allows researchers, scientists, or readers to interpret them more easily. These indicators differ depending on the life cycle impact assessment (LCIA) method selected. The results of each process are shown as a list of emissions and consumed resources, and the LCIA methods distribute the emissions and consumed resources in a shorter list of indicators.

The LCIA method chosen is the ReCiPe method. There are two main impact assessment approaches, the midpoint and the endpoint, and the LCIA method transforms the emissions and the resource consumptions into an indicator, depending on the approach. For example, CML is an LCIA method that gives a midpoint approach, a list of indicators that shows a complete environmental profile that is difficult to interpret [58]. On the other hand, the eco-indicator LCIA method gives an endpoint approach. This approach takes the midpoint approach indicators and concentrates them in three damage categories: resources measured in dollars, human health measured in disability-adjusted life years, and ecosystem impact measured in species·year. This endpoint approach allows researchers to analyze the impact of the activity more easily. The ReCiPe LCIA method provides both the endpoint and the midpoint approaches and has therefore been chosen for the LCIA.

The ReCiPe midpoint approach provides a list of 18 environmental indicators. These indicators are useful if the study carried out is focused on one specific impact, such as the global warming potential or the metal depletion. The categories supplied by this method are: agricultural land occupation (ALO), global warming potential (GWP), fossil depletion (FD), freshwater ecotoxicity (FEPT), freshwater eutrophication (FEP), human toxicity (HTP), ionizing radiation (IRP), marine ecotoxicity (MEPT), marine eutrophication (MEP), metal depletion (MD), natural land transformation (NLT), ozone depletion (OD), particulate matter formation (PMF), photochemical oxidant formation (POFP), terrestrial acidification (TAP), terrestrial ecotoxicity (TEPT), urban land occupation (ULO), and water depletion (WD). In this study, the recycling and further use of the materials has been considered and therefore the hierarchist (H) version is chosen [59]. To assess the total impact, the normalization of the endpoint impact is needed in order to add the three categories. The normalization set used in this research is the Europe ReCiPe H/A person/year.

### 2.6. Interpretation

The interpretation phase is the last stage of the LCA, in which the impact results of the analyzed activity are evaluated and compared with other activities or studies. The interpretation depends on the objective of the study. The study can be focused on the contribution of each life cycle phase to the final impact, on the impact of every material compared with the others, or on the comparison between the emissions or the resource consumption between alternatives, among others. In this study, the comparison between the different bridge deck alternatives is carried out.

## 3. Life Cycle Assessment

**Figure 3:**
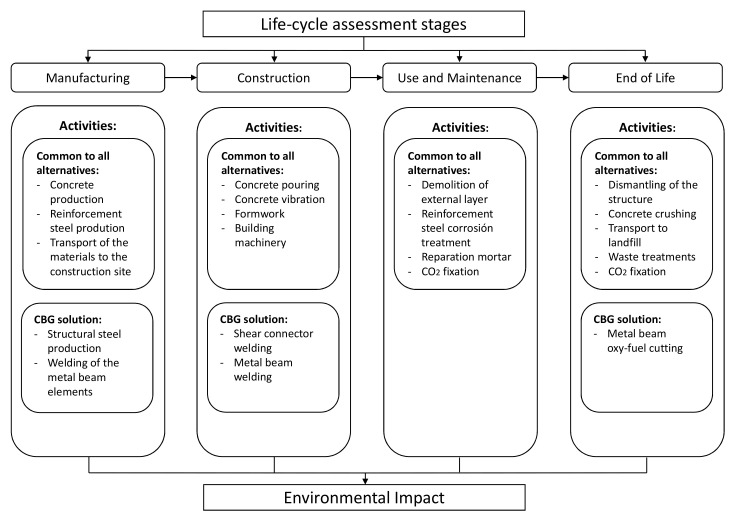
Life cycle of the bridge decks.

In this research, the uncertainty has been considered using a Monte Carlo simulation with 1000 iterations to obtain the probabilistic uncertainty values of the LCA results. In the comparison graphs, only the mean values are shown to make them easier to interpret. The life cycle flowchart for bridge decks is summarized in Figure 3.

### 3.1. Midpoint Approach

**Figure 4:**
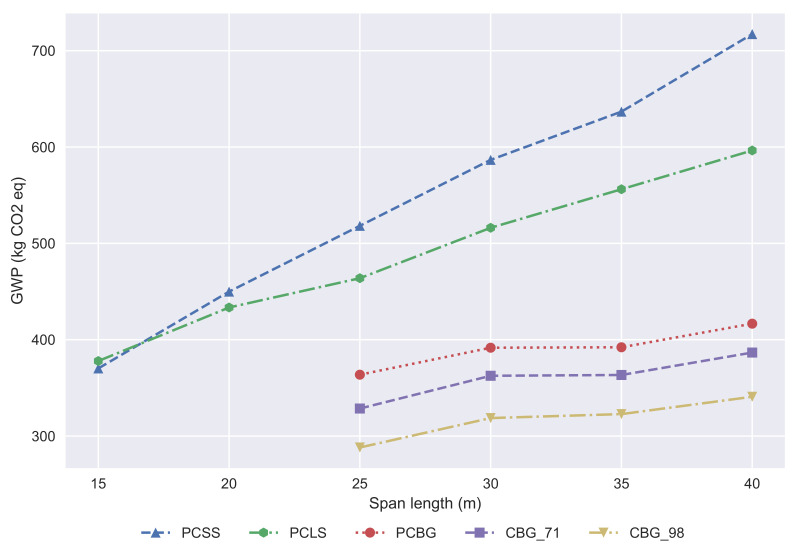
Development of GWP according to the span length.

The midpoint impact categories, as stated before, provide more reliable results due to the wide range of indicators provided. The data obtained allows the study to be focused on particular impacts, such as the global warming potential, evaluating the CO_2_ emissions of the activity. The full results of a 35 m span length bridge are provided in Table 3, including the coefficients of variation of all indicators. A global warming potential (GWP) study has been done to compare the emissions of each alternative, as shown in Figure 4. The difference between the slab decks and the box-girder can be clearly distinguished. For span lengths in which the box-girder decks are built, these are the best alternatives from the GWP point of view. The PCLS emits lower quantities of CO_2_ than the PCSS for span lengths of less than 17 m. In larger span length ranges, we can state that the CBG bridges are better than the PCBG, even though we consider a 71% steel recycling ratio for the structural steel (CBG_71). If we focus on the contribution of every life cycle stage to the GWP indicator, it is observed that in the end of life phase, all the alternatives have a negative impact on the GWP, due to the CO_2_ fixation caused by the carbonation of concrete.

**Figure 5:**
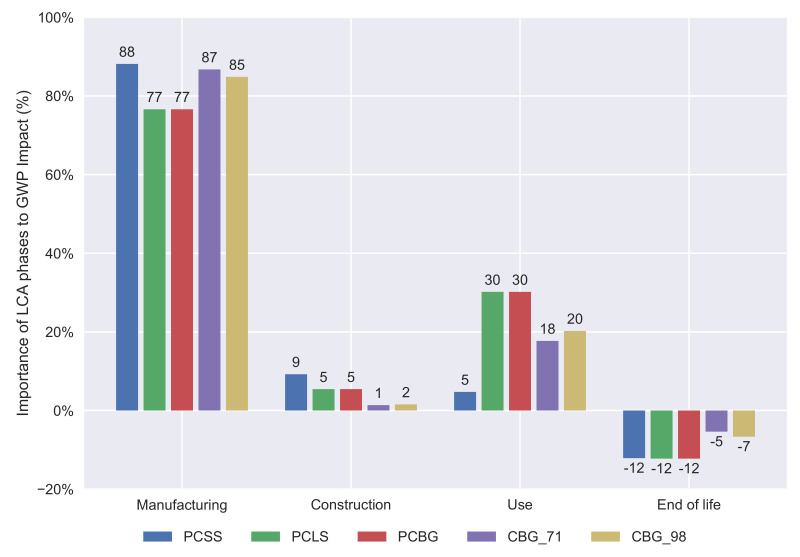
Contribution of deck alternatives to life cycle stages for 35 m span length.

Figure 5 shows that the concrete alternatives (PCSS, PCLS, and PCBG) have a greater negative impact in the end of life phase, because the high amounts of concrete that they contain allow a great CO_2_ fixation by the carbonation processes. The PCBG alternative has a greater impact on the GWP during the use and maintenance phase because it has a larger surface to repair.

**Figure 6:**
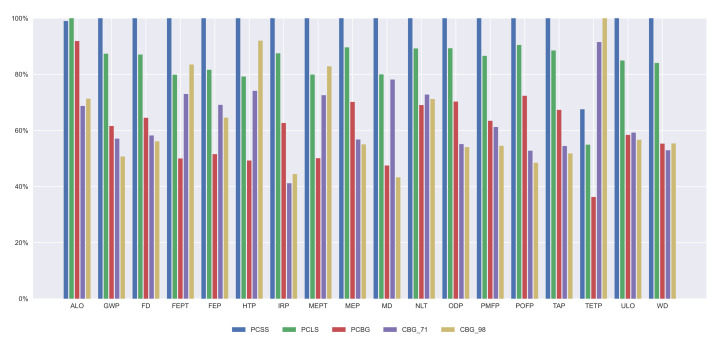
Midpoint impacts for 35 m span length.

A comparison between all the alternatives for every midpoint approach impact is presented in Figure 6 for a 35 m span length bridge, providing their impact relative to the biggest one. The alternative that has the most impact in all the categories is the PCSS, excluding the TEP where the highest impact alternative is the CBG_98. In MD, the CBG_71 reaches the impact of the PCLS alternative because of the large amount of steel that is needed for this bridge deck section. However, the CBG_91 alternative, from which a high impact was expected, does not produce such a high one because of the steel recycling, which allows for the generation of a new product with the same characteristics using low amounts of raw material. These steel recycling processes, excluding the raw material reduction, produce a greater impact on the HTP indicator.

**Figure 7:**
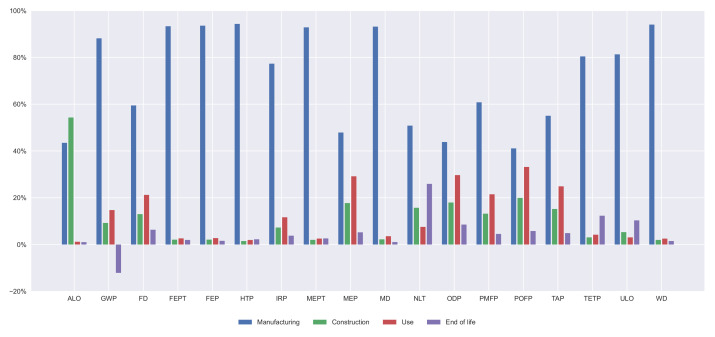
Impact categories for 35 m span length PCSS solution.

**Figure 8:**
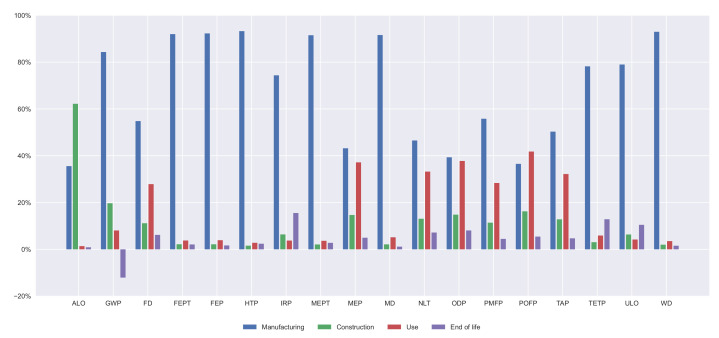
Impact categories for 35 m span length PCLS solution.

**Figure 9:**
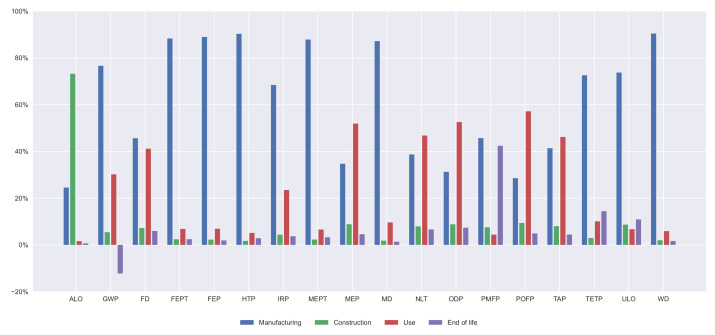
Impact categories for 35 m span length PCBG solution.

**Figure 10:**
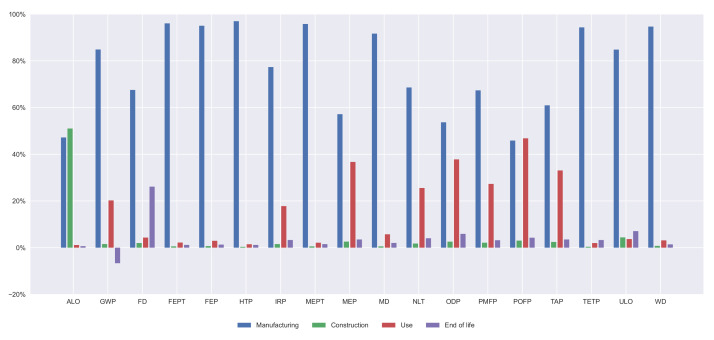
Impact categories for 35 m span length CBG_98 solution.

Furthermore, the contribution of every life cycle stage on every indicator is illustrated in Figure 7, Figure 8, Figure 9 and Figure 10. In all the alternatives, the life cycle phase that has the highest impact in most categories is manufacturing, but there are many exceptions. For the ALO indicator, the phase with the greatest impact is the construction for all the alternatives. The contribution of the use and maintenance stage has a greater impact in the MEP, NLT, ODP, PMFP, POFP, and TAP indicators, especially in the PCBG bridge deck alternative, due to having the highest surface exposed to the environmental conditions, which requires more maintenance. In Figure 7, Figure 8, Figure 9 and Figure 10, the results of the midpoint approach for PCSS, PCLS, PCBG, and CBG_98 are shown.

### 3.2. Endpoint Approach

To obtain the assessment results in a way that is easier to interpret to compare between different categories, the endpoint approach is provided. The three categories can be significant for choosing the best alternative depending on the situation. If the study area is close to a protected area, then the environmental impact of the structure will be the most important one for the study. If it is built close to a population center, the human health impact will be the most significant, and if the location lacks resources, then the resources category will be the most important one. In this study, a normalization set has been applied to these three impact category results to obtain a global impact. This is useful when there is no preference between the environmental criteria, and equal importance is considered for all the criteria. In this way, a total impact score for the bridge deck alternatives was obtained. The normalization and weighting set adopted was the Europe ReCiPe H/A person/year.

A comparison between the PCSS, PCLS, PCBG, and CBG bridge deck solutions was done. For the CBG alternative, three structural steel recycling ratios were considered, 71% (CBG_71), 90% (CBG_90), and 98% (CBG_98), to analyze the differences between the composite box-girder bridge decks and the concrete alternatives.

**Figure 11:**
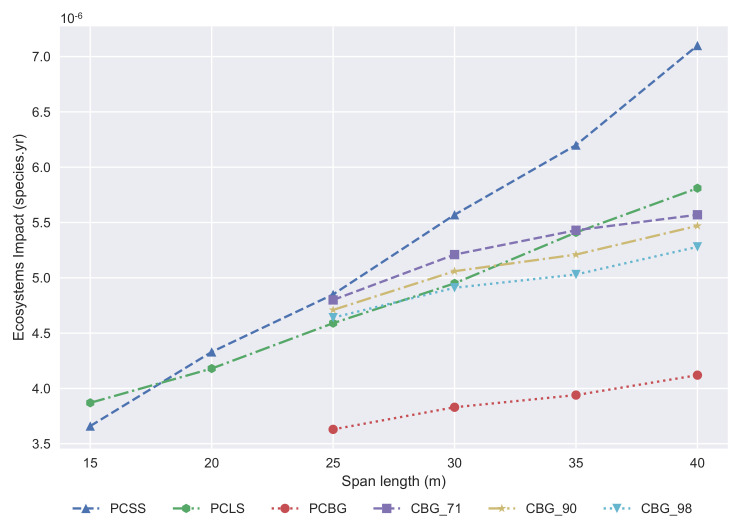
Development of the ecosystems impact with regard to span length.

First, the ecosystems impact is provided in Figure 11 in species.year. The best solution is the PCBG, the PCSS is competitive for span lengths shorter than 18 meters, and for longer ones, the PCLS is even better than the CBG alternative. If the steel recycling ratio of the structural steel is 98%, then the PCLS is better until 30 m, but if we reduce that ratio, then the PCLS is the best solution until 35 m. These results are because the PCSS needs great amounts of steel when the span length increases. If we compare the PCLS and the CBG, the high increment of materials for the PCLS is compensated by the high environmental cost of the steel production when the recycling ratio of this process is lower.

**Figure 12:**
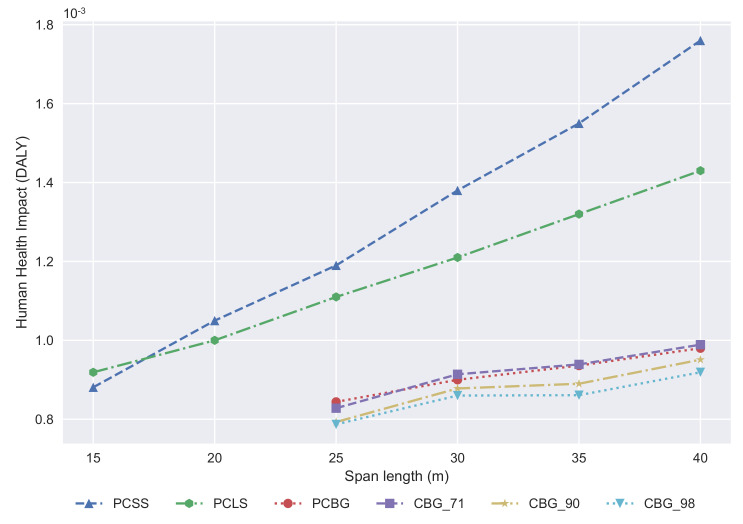
Development of the human health impact with regard to span length.

The damage caused to human health is measured in disability-adjusted life years, and it is shown in Figure 12. PCSS is a competitive alternative up to 17 m, then the PCLS is the best alternative from that span length up to 25 m. The PCBG alternative is competitive with CBG when the structural steel recycling ratio is 71%. If the recycling ratio is greater, then the CBG alternative is the best solution.

**Figure 13:**
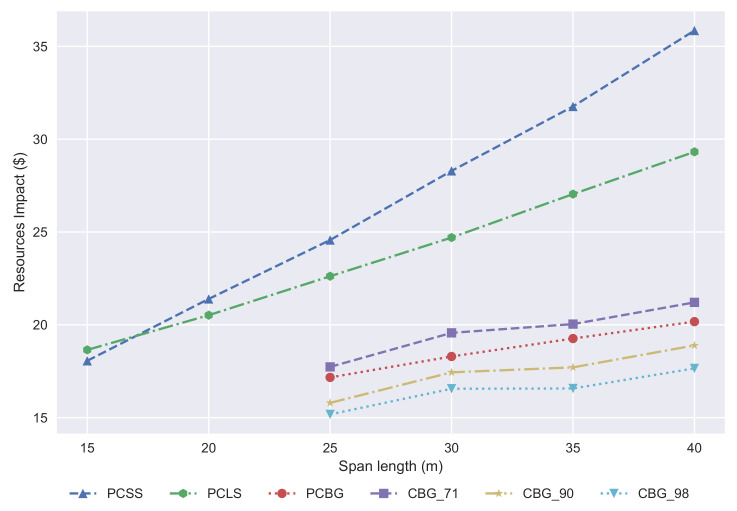
Development of the resources impact with regard to span length.

The damage caused by the resources, measured in dollars, is shown in Figure 13. The PCSS remains the best solution for span lengths shorter than 17 m, and from there up to 25 m, the best solutions are the box-girders. From the resources point of view, the best solution is the CBG if the structural steel recycling ratio is 90%; if it is not, then the PCBG is a competitive solution compared with the CBG.

**Figure 14:**
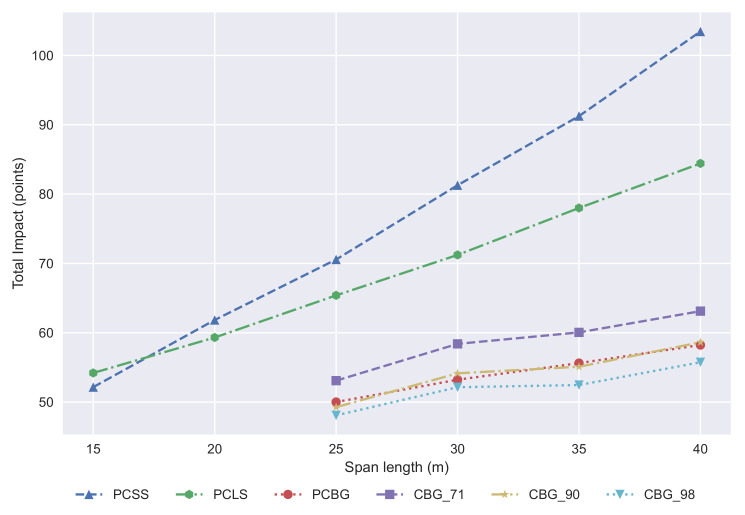
Development of the total impact with regard to span length.

Normalization has been done to compare the total impact results. These results are provided in Figure 14 measured in points. The best solutions are usually the CBG with structural steel recycling ratios higher than 90% in the ranges where they are usually built. If the ratio is lower, then the PCBG is the best solution. The PCSS and PCLS solutions are much worse in terms of the environmental impact due to the great increase of materials as the span length increases. These alternatives must be used for lengths less than 25 m from the environmental point of view, and, tuning even more, the PCSS alternative is the most sustainable one for span lengths below 17 m.

**Figure 15:**
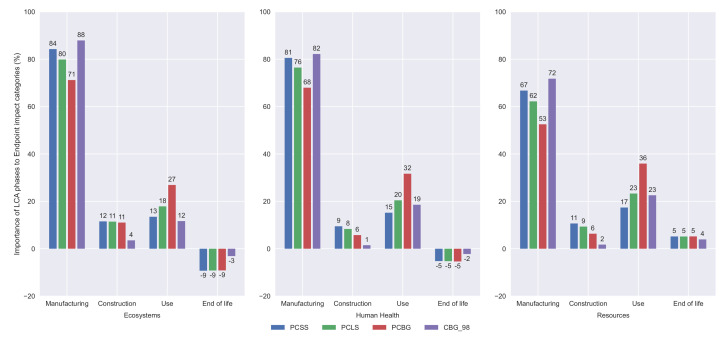
Importance of the LCA stages according to the endpoint impact categories.

A comparison between the contributions of the life cycle stages considered in this study is shown in Figure 15 for the three main environmental categories. For all categories, the manufacturing process is the most important one and the construction the least. It is observed that in the end of life stage, the concrete carbonation of the crushed concrete gives a negative value, which becomes even lower for the CBG_98 alternative, due to the smaller amounts of concrete used for this bridge deck section. The use and maintenance stage is more important for the PCBG alternative. The contribution of the construction phase is almost null for the composite alternative in all categories.

**Figure 16:**
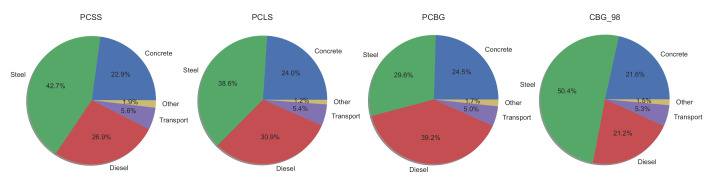
Importance of materials to total impact.

Finally, a comparison between the impacts of every material on the total impact is provided in Figure 16. Steel and concrete are the most important materials in terms of the environmental impact. In the PCBG, the diesel consumption also becomes important. It is observed that the contribution of steel takes a value of 50.44% for the composite alternative.

## 4. Conclusions

One of the most important sectors that influences climate change is construction. For this reason, environmental assessments are required to analyze the impact of construction and to select options that do not affect the future of the planet. In this study, an LCA has been done for four different bridge deck sections, with an endpoint and midpoint approach.

A comparison between the impacts of the alternatives has been carried out. For span lengths less than 25 m, the box-girder solutions have not generally been used, but they are the best alternatives in terms of the environmental impact for these lengths. The PCSS and PCLS alternatives are competitive from 15 to 25 m, and between 15 and 17 m, the best solution is the PCSS. The difference between these slab bridge deck alternatives is caused by the increment of materials for the PCSS alternative, mainly steel.

The steel recycling ratio is determinant in comparing steel and composite bridge decks. The structural recycling ratio is usually greater than the rebar one, and this difference is reflected in the impact values. If we consider the same steel recycling ratio for structural and rebar steel, the box-girder concrete alternatives are better than the composite ones. If we consider a 98% steel recycling ratio, this being the value in the USA, then the best alternative is the CBG.

The consideration of CO_2_ fixation by carbonation processes has an important impact on the evaluation and comparison of the alternatives, because the composite structures use a smaller amount of concrete, which is reflected as lower CO_2_ emissions in the environmental impact assessment. For concrete structures, the carbonation processes, even though they are negative for the steel reinforcements, make concrete solutions more competitive than the steel structures.

The most important LCA phase for all the alternatives is the manufacturing, and for the concrete alternatives (PCSS, PCLS, and PCBG), the use and maintenance phase has a great impact due to the greater surface of concrete that has to be maintained. In this study, the steel does not require maintenance because it is a weathering steel. In further studies, the steel maintenance can be considered. The concrete maintenance activities are an important factor in the impact; if the durability of the material increases, then this maintenance will be reduced.

Every graph and result shown in this paper will help engineers, designers, and constructors to select cleaner alternatives, and if they consider their own countries’ material production processes, they can choose the best environmental solution. For countries where there is less steel recycling, concrete structures will be the best solution, but in countries with cleaner steel production processes, composite and steel solutions will be the best ones from the environmental point of view.

## Figures and Tables

**Table 1:** Amount of materials per square meter of deck.

	Unit	15	20	25	30	35	40
**PCSS**							
Concrete HP-40	m³	0.473	0.561	0.649	0.738	0.826	0.914
Reinforcement Steel	kg	51.728	61.380	71.033	80.686	90.339	99.992
Prestressed Reinforcement Steel	kg	9.223	17.133	25.043	32.953	40.863	48.773
Formwork	m²	1.500	1.500	1.500	1.500	1.500	1.500
**PCLS**							
Concrete HP-40	m³	0.509	0.557	0.605	0.654	0.702	0.750
Reinforcement Steel	kg	52.165	57.109	62.052	66.996	71.939	76.883
Prestressed Reinforcement Steel	kg	5.069	10.914	16.759	22.604	28.449	34.294
Formwork	m²	1.700	1.700	1.700	1.700	1.700	1.700
**PCBG**							
Concrete HP-40	m³	0.441	0.461	0.482	0.503	0.523	0.544
Reinforcement Steel	kg	28.790	32.601	36.632	40.884	45.356	50.048
Prestressed Reinforcement Steel	kg	3.042	4.917	6.792	8.667	10.542	12.417
Formwork	m²	1.900	1.900	1.900	1.900	1.900	1.900
**CBG**							
Concrete HA-30	m³	0.220	0.230	0.240	0.250	0.261	0.272
Reinforcement Steel	kg	20.976	22.250	23.603	25.037	26.559	28.173
Structural Steel	kg	59.400	63.700	68.175	81.000	80.600	88.375
Shear Connector Steel	kg	0.310	0.346	0.381	0.423	0.437	0.494
Formwork	m²	1.000	1.000	1.000	1.000	1.000	1.000

**Table 2:** Concrete dosage considered for bridge decks.

		Concrete	
Material	Unit	HA-30	HP-40
Gravel	kg	1110.00	829.00
Sand	kg	730.00	1102.00
Cement	kg	300.00	320.00
Water	kg	201.00	160.00
Superplasticizer	kg	0.27	5.00

**Table 3:** Midpoint approach impacts of 35 m long bridges. Mean and coefficient of variation (cv).

		PCSS		PCLS		PCBG		CBG_98	
Acronym	Unit	Mean	cv (%)	Mean	cv (%)	Mean	cv (%)	Mean	cv (%)
**ALO**	m2*a	31.35	59.31%	31.67	63.27%	29.09	71.18%	22.583	55.17%
GWP	kg CO_2_ eq	636.76	45.72%	556.19	44.00%	392.14	40.28%	322.776	35.37%
FD	kg oil eq	148.79	26.09%	129.51	24.25%	95.97	20.58%	83.494	26.26%
FEPT	kg 1,4-DB eq	7.53	41.45%	6.01	40.54%	3.76	39.15%	6.285	43.37%
FEP	kg P eq	0.16	40.84%	0.13	39.08%	0.08	37.43%	0.100	37.76%
HTP	kg 1,4-DB eq	276.00	44.56%	218.63	44.03%	135.98	42.66%	253.954	47.37%
IRP	kg U235 eq	56.39	44.71%	49.34	44.12%	35.33	40.17%	25.067	31.96%
MEPT	kg 1,4-DB eq	7.41	40.88%	5.92	39.98%	3.71	38.60%	6.140	43.13%
MEP	kg N eq	0.14	23.59%	0.13	21.70%	0.10	17.53%	0.079	24.98%
MD	kg Fe eq	98.26	50.78%	78.60	47.04%	46.66	44.54%	42.531	35.66%
NLT	m2	0.13	25.82%	0.12	24.29%	0.09	20.45%	0.094	39.37%
ODP	kg CFC-11 eq	0.00	19.32%	0.00	17.68%	0.00	14.55%	0.000	21.31%
PMFP	kg PM10 eq	1.74	27.47%	1.51	24.64%	1.10	20.31%	0.948	26.43%
POFP	kg NMVOC	3.63	19.95%	3.29	17.87%	2.63	14.03%	1.761	18.52%
TAP	kg SO2 eq	2.90	26.53%	2.57	24.56%	1.95	20.35%	1.504	24.73%
TETP	kg 1,4-DB eq	0.08	33.95%	0.07	33.60%	0.04	32.70%	0.122	48.71%
ULO	m2*a	7.18	34.96%	6.10	34.11%	4.19	33.32%	4.070	32.24%
WD	m3	1540.31	45.90%	1294.58	45.72%	851.61	44.25%	852.564	38.79%

## Data Availability

The results of the experiments are in: https://drive.google.com/file/d/1yh7IQarxaA8Iht-YKKPtSre32hbQXl6_/view?usp=sharing (accessed on 27 July 2021).

## Abbreviations

The following abbreviations are used in this manuscript:

LCA	Life Cycle Assessment
LCIA	LIfe Cycle Inventory Assessment
PCSS	Prestressed Concrete Solid Slab
PCLS	Prestressed Concrete Lightened Slab
PCBG	Prestressed Concrete Box Girder
CBG	Composite Box Girder
PRS	Passive Reinforcing Steel
ARS	Active Reinforcing Steel
BOF	Basic Oxygen Furnace
EAF	Electric Arc Furnace
ALO	Agricultural Land Occupation
GWP	Global Warming Potential
FD	Fossil Depletion
FEPT	Freshwater Ecotoxicity
FEP	Freshwater Eutrophication
HTP	Human Toxicity
IRP	Ionizing Radiation
MEPT	Marine Ecotoxicity
MEP	Marine Eutrophication
MD	Metal Depletion
NLT	Natural Land Transformation
OD	Ozone Depletion
PMF	Particule Matter Formation
POFP	Photochemical Oxidant Formation
TAP	Terrestrial Acidification
TEPT	Terrestrial Ecotoxicity
ULO	Urban Land Occupation
WD	Water Depletion

## References

[1] WCED (1987). Our Common Future.

[2] Petek Gursel A., Masanet E., Horvath A., Stadel A. (2014). Life-cycle inventory analysis of concrete production: A critical review. Cem. Concr. Compos..

[3] Ramesh T., Prakash R., Shukla K.K. (2010). Life cycle energy analysis of buildings: An overview. Energy Build..

[4] Årskog V., Fossdal S., Gjørv O.E. Life-Cycle Asessment of Repair and Maintenance Systems for Concrete Structures. Proceedings of the International Workshop on Sustainable Development and Concrete Technology.

[5] Shen L.Y., Lu W., Yao H., Wu D.H. (2005). A computer-based scoring method for measuring the environmental performance of construction activities. Autom. Constr..

[6] Boesch M.E., Hellweg S. (2010). Identifying improvement potentials in cement production with life cycle assessment. Autom. Constr..

[7] Taylor M., Tam C., Gielen D. (2006). Energy Efficiency and CO_2_ Emissions from the Global Cement Industry, Energy Technology Policy Division.

[8] Collins F. (2010). Inclusion of carbonation during the life cycle of built and recycled concrete: Influence on their carbon footprint. Int. J. Life Cycle Assess..

[9] García-Segura T., Yepes V., Frangopol D.M. (2017). Multi-objective design of post-tensioned concrete road bridges using artificial neural networks. Struct. Multidiscip. Optim..

[10] Yepes V., Martí J.V., García-Segura T. (2015). Cost and CO_2_ emission optimization of precast-prestressed concrete U-beam road bridges by a hybrid glowworm swarm algorithm. Autom. Constr..

[11] Serpell A., Kort J., Vera S. (2013). Awareness, actions, drivers and barriers of sustainable construction in chile. Technol. Econ. Dev. Econ..

[12] Sierra L.A., Pellicer E., Yepes V. (2017). Method for estimating the social sustainability of infrastructure projects. Environ. Impact Assess. Rev..

[13] Yusof N., Zainul Abidin N., Zailani S.H.M., Govindan K., Iranmanesh M. (2016). Linking the environmental practice of construction firms and the environmental behaviour of practitioners in construction projects. J. Clean. Prod..

[14] García-Segura T., Yepes V., Alcala J., Pérez-López E. (2015). Hybrid harmony search for sustainable design of post-tensioned concrete box-girder pedestrian bridges. Eng. Struct..

[15] Marti J.V., Yepes V., Gonzalez-Vidosa F. (2015). Memetic Algorithm Approach to Designing Precast-Prestressed Concrete Road Bridges with Steel Fiber Reinforcement. J. Struct. Eng..

[16] Molina-Moreno F., García-Segura T., Martí J.V., Yepes V. (2017). Optimization of buttressed earth-retaining walls using hybrid harmony search algorithms. Eng. Struct..

[17] Martínez-Muñoz D., Martí J.V., García J., Yepes V. (2021). Embodied Energy Optimization of Buttressed Earth-Retaining Walls with Hybrid Simulated Annealing. Appl. Sci..

[18] Yepes V., Alcalá J., Perea C., González-Vidosa F. (2008). A parametric study of optimum earth-retaining walls by simulated annealing. Eng. Struct..

[19] Yepes V., González-Vidosa F., Alcalá J., Villalba P. (2012). CO_2_-Optimization Design of Reinforced Concrete Retaining Walls Based on a VNS-Threshold Acceptance Strategy. J. Comput. Civ. Eng..

[20] Dodoo A., Gustavsson L., Sathre R. (2009). Carbon implications of end-of-life management of building materials. Resour. Conserv. Recycl..

[21] García-Segura T., Yepes V., Alcalá J. (2014). Life cycle greenhouse gas emissions of blended cement concrete including carbonation and durability. Int. J. Life Cycle Assess..

[22] Du G., Karoumi R. (2013). Life cycle assessment of a railway bridge: Comparison of two superstructure designs. Struct. Infrastruct. Eng..

[23] Du G., Safi M., Pettersson L., Karoumi R. (2014). Life cycle assessment as a decision support tool for bridge procurement: Environmental impact comparison among five bridge designs. Int. J. Life Cycle Assess..

[24] Hettinger A., Birat J., Hechler O., Braun M. (2015). Sustainable Bridges—LCA for a Composite and a Concrete Bridge.

[25] Salvador R., Barros M., dos Santos G., van Mierlo K., Piekarski C., de Francisco A. (2021). Towards a green and fast production system: Integrating life cycle assessment and value stream mapping for decision making. Environ. Impact Assess. Rev..

[26] Vitale P., Napolitano R., Colella F., Menna C., Asprone D. (2021). Cement-Matrix Composites Using CFRP Waste: A Circular Economy Perspective Using Industrial Symbiosis. Materials.

[27] Caneda-Martínez L., Monasterio M., Moreno-Juez J., Martínez-Ramírez S., García R., Frías M. (2021). Behaviour and Properties of Eco-Cement Pastes Elaborated with Recycled Concrete Powder from Construction and Demolition Wastes. Materials.

[28] Jiang Q., Wang F., Liu Q., Xie J., Wu S. (2021). Energy Consumption and Environment Performance Analysis of Induction-Healed Asphalt Pavement by Life Cycle Assessment (LCA). Materials.

[29] Zastrow P., Molina-Moreno F., García-Segura T., Martí J., Yepes V. (2017). Life cycle assessment of cost-optimized buttress earth-retaining walls: A parametric study. J. Clean. Prod..

[30] Pons J.J., Penadés-Plà V., Yepes V., Martí J.V. (2018). Life cycle assessment of earth-retaining walls: An environmental comparison. J. Clean. Prod..

[31] Penadés-Plà V., Martínez-Muñoz D., García-Segura T., Navarro I., Yepes V. (2020). Environmental and Social Impact Assessment of Optimized Post-Tensioned Concrete Road Bridges. Sustainability.

[32] Sánchez-Garrido A., Navarro I., Yepes V. (2021). Neutrosophic multi-criteria evaluation of sustainable alternatives for the structure of single-family homes. Environ. Impact Assess. Rev..

[33] Laiblová L., Pešta J., Kumar A., Hájek P., Fiala C., Vlach T., Kočí V. (2019). Environmental Impact of Textile Reinforced Concrete Facades Compared to Conventional Solutions—LCA Case Study. Materials.

[34] Kvočka D., Lešek A., Knez F., Ducman V., Panizza M., Tsoutis C., Bernardi A. (2020). Life Cycle Assessment of Prefabricated Geopolymeric Façade Cladding Panels Made from Large Fractions of Recycled Construction and Demolition Waste. Materials.

[35] Martínez-Muñoz D., Martí J.V., Yepes V. (2020). Steel-Concrete Composite Bridges: Design, Life Cycle Assessment, Maintenance, and Decision-Making. Adv. Civ. Eng..

[36] Rossi B., Marquart S., Rossi G. (2017). Comparative life cycle cost assessment of painted and hot-dip galvanized bridges. J. Environ. Manag..

[37] Worldsteel (2017). Life Cycle Inventory Methodology Report for Steel Products.

[38] ISO (2006). Environmental Management, Life Cycle Assessment Principles and Framework (ISO 14040:2006).

[39] Goedkoop M., Heijungs R., Huijbregts M., De Schryver A., Struijs J., Van Zelm R. (2009). ReCiPe 2008. Report I: Characterisation.

[40] Pang B., Yang P., Wang Y., Kendall A., Xie H., Zhang Y. (2015). Life cycle environmental impact assessment of a bridge with different strengthening schemes. Int. J. Life Cycle Assess..

[41] Marceau M., Nisbet M., Vangeem M. (2002). Life Cycle Inventory of Portland Cement Concrete.

[42] SRI Construction | SRI—Steel Recycling Institute. https://www.steelsustainability.org/construction.

[43] Hammervold J., Reenaas M., Brattebø H. (2013). Environmental Life Cycle Assessment of Bridges. J. Bridge Eng..

[44] García-Segura T., Yepes V. (2016). Multiobjective optimization of post-tensioned concrete box-girder road bridges considering cost, CO_2_ emissions, and safety. Eng. Struct..

[45] Lagerblad B. (2005). Carbon Dioxide Uptake during Concrete Life Cycle—State of the Art.

[46] Penadés-Plà V., Martí J.V., García-Segura T., Yepes V. (2017). Life-Cycle Assessment: A Comparison between Two Optimal Post-Tensioned Concrete Box-Girder Road Bridges. Sustainability.

[47] MFOM (2011). Instrucción de Hormigón Estructural (EHE-08).

[48] Ciroth A. (2007). ICT for environment in life cycle applications openLCA—A new open source software for Life Cycle Assessment. Int. J. Life Cycle Assess..

[49] Frischknecht R., Rebitzer G. (2005). The ecoinvent database system: A comprehensive web-based LCA database. J. Clean. Prod..

[50] Pascual-González J., Guillén-Gosálbez G., Mateo-Sanz J.M., Jiménez-Esteller L. (2016). Statistical analysis of the ecoinvent database to uncover relationships between life cycle impact assessment metrics. J. Clean. Prod..

[51] Larsen S. (2021). Inclusion of uncertainty in Environmental Impact Assessment in Greenland. Environ. Impact Assess. Rev..

[52] Hong J., Shen G.Q., Peng Y., Feng Y., Mao C. (2017). Reprint of: Uncertainty analysis for measuring greenhouse gas emissions in the building construction phase: A case study in China. J. Clean. Prod..

[53] Ciroth A., Muller S., Weidema B., Lesage P. (2016). Empirically based uncertainty factors for the pedigree matrix in ecoinvent. Int. J. Life Cycle Assess..

[54] Yepes V., Díaz J., González-Vidosa F., Alcalá J. (2009). Statistical Characterization of Prestressed Concrete Road Bridge Decks. Rev. Construcción.

[55] MFOM (2000). Obras de Paso de Nueva Construcción.

[56] Zhang Y., Mao Y., Jiao L., Shuai C., Zhang H. (2021). Eco-efficiency, eco-technology innovation and eco-well-being performance to improve global sustainable development. Environ. Impact Assess. Rev..

[57] Catalonia Institute of Construction Technology BEDEC ITEC Materials Database. https://metabase.itec.cat/vide/es/bedec.

[58] Yi S., Kurisu K., Hanaki K. (2011). Life cycle impact assessment and interpretation of municipal solid waste management scenarios based on the midpoint and endpoint approaches. Int. J. Life Cycle Assess..

[59] Khatri P., Jain S., Pandey S. (2017). A cradle-to-gate assessment of environmental impacts for production of mustard oil using life cycle assessment approach. J. Clean. Prod..

